# Retrospective analysis of dietary risk factors and cardiovascular outcomes in angiocardiopathy

**DOI:** 10.3389/fnut.2026.1780549

**Published:** 2026-03-19

**Authors:** Hongtao Yin, Yanqing Zhou, Yingjun Zhou, Lin Ren, Lixiang Ma

**Affiliations:** 1 Department of Cardiology, The First Hospital of Qinhuangdao, Qinhuangdao, Hebei, China; 2 Department of Operating Room, The First Hospital of Qinhuangdao, Qinhuangdao, Hebei, China

**Keywords:** angiocardiopathy, cardiovascular disease risk, dietary risk factors, nutritional epidemiology, retrospective analysis

## Abstract

**Background:**

Angiocardiopathy, encompassing structural and functional heart and vascular disorders, is associated with multiple modifiable risk factors, among which diet plays a pivotal role.

**Objective:**

This study aimed to examine the association between dietary risk patterns and cardiovascular events in patients with angiocardiopathy.

**Methods:**

This retrospective cross-sectional study involved 525 patients who were categorized into three groups: low (*n* = 143), moderate (*n* = 216), and high (*n* = 166) dietary risk groups based on their dietary intake and the Dietary Inflammatory Index (DII) scores. Information on sociodemographic factors, anthropometry, clinical status, diet, and cardiovascular outcomes was collected from medical records and analyzed. Multivariable logistic regression was used to compute adjusted odds ratios (ORs) with 95% confidence intervals (CIs).

**Results:**

Patients in the high dietary risk group were generally older (60.1 ± 13.1 vs. 56.2 ± 11.8 years, *p* = 0.021), with a higher proportion having lower education (no formal education: 33.1% vs. 15.4%, *p* = 0.015) and lower income (54.8% vs. 33.6%, *p* = 0.009). Anthropometric measures, including BMI (28.7 ± 5.2 vs. 26.6 ± 4.4 kg/m^2^, *p* = 0.002) and waist circumference (97.6 ± 12.1 vs. 91.2 ± 10.5 cm, *p* < 0.001), were also higher in the high-risk group. These individuals consumed more energy (2,471 ± 425 kcal/day), saturated fat (14.8 ± 2.6% of energy), and sodium (3,560 ± 640 mg/day), and had higher DII scores (3.4 ± 1.1), while fiber (14.6 ± 3.9 g/day) and omega-3 intake (0.6 ± 0.3 g/day) were lower (all *p* < 0.001). The prevalence of cardiovascular events was markedly higher in the high-risk group (73.5%) compared to the low-risk group (37.8%, *p* < 0.001). High dietary risk was significantly associated with MACE (OR 2.63, 95% CI 1.81–3.83), stroke (OR 4.82, 95% CI 2.41–9.64), and cardiovascular mortality (OR 4.13, 95% CI 1.78–9.57).

**Conclusion:**

Pro-inflammatory, high-risk dietary patterns were significantly associated with a higher prevalence of adverse cardiovascular outcomes in patients with angiocardiopathy.

## 1 Introduction

Angiocardiopathy refers to a spectrum of heart and vascular disorders that encompass structural, functional, and circulatory abnormalities. It includes conditions such as ischemic heart disease, heart failure, arrhythmias, and vascular dysfunction that compromise cardiovascular health (1), is a major public health concern due to its close association with adverse cardiovascular outcomes (2). For patient inclusion in studies, angiocardiopathy was defined using a combination of standardized clinical codes and objective diagnostic criteria. Specifically, patients were identified using ICD-10 codes I00–I99, encompassing a broad spectrum of cardiovascular diseases. To ensure clinical specificity, objective measures were also applied, including echocardiographic evidence of structural or functional cardiac abnormalities (e.g., left ventricular ejection fraction [LVEF] < 50%, moderate-to-severe valvular lesions) or angiographic confirmation of significant coronary or peripheral vascular pathology. From an epidemiological perspective, cardiovascular diseases are responsible for around one-third of deaths worldwide, and a large portion of this health burden is statistically attributed to dietary risk factors (3, 4). Dietary behaviors can be classified as low, moderate, or high risk based on their adherence to nutritional guidelines and their inflammatory potential, which is most often assessed by the Dietary Inflammatory Index (DII) (5). People in the low-risk group normally have a diet that consists of an adequate number of fruits, vegetables, whole grains, dietary fiber, omega-3 fatty acids, and micronutrients, and at the same time, they limit the intake of added sugars, saturated/trans fats, and sodium (6, 7). This corresponds to lower DII scores (1.0) and a mainly anti-inflammatory dietary pattern. On the other hand, moderate-risk diets (DII 1.1 ± 2.9) can be described as partially following healthy eating patterns with moderate consumption of fruits and vegetables (8), higher consumption of refined carbohydrates, and slightly elevated intake of saturated fat, sugar, or sodium (9). High-risk dietary patterns (DII 3.0) are characterized by a lower intake of fruits, vegetables, and fiber is very low, and there is excessive consumption of added sugars, saturated and trans fats, and sodium (10), reflecting a more pro-inflammatory dietary profile. This indicates a highly pro-inflammatory nutritional environment. Large cohort and population-based studies have consistently reported significant associations between unhealthy, pro-inflammatory dietary patterns and a higher incidence and progression of angio-cardiopathic conditions (11, 12). These dietary patterns have been associated with adverse cardiometabolic profiles, including endothelial dysfunction, dyslipidemia, hypertension, insulin resistance, and chronic low-grade inflammation (13). Proposed mechanisms underlying these associations include pathways related to oxidative stress, reduced nitric oxide bioavailability, activation of pro-inflammatory cytokines, and enhanced lipid oxidation, which have been correlated with atherogenesis, vascular remodeling, myocardial structural alterations, and impaired perfusion (14). Conversely, cardioprotective dietary components have been associated with more favorable vascular homeostasis and lower cardiovascular risk profiles (15). Within this framework, retrospective analyses integrating dietary risk stratification with cardiovascular outcomes provide an opportunity to explore associations between long-term dietary exposures and clinical trajectories, thereby contributing to the understanding of population-level patterns linking diet and cardiovascular health (16, 17).

Diet has been acknowledged as a major factor in disease progression and cardiovascular outcomes. While strong associations between dietary patterns and cardiovascular disease have been confirmed, there is still a scarcity of robust evidence that specifically points to the dietary intake of patients with angiocardiopathy. Retrospective study designs enable the evaluation of cumulative dietary exposures in real-world populations and their statistical associations with major cardiovascular outcomes. Diets characterized by higher intake of saturated and trans fats, sodium, and refined carbohydrates have been associated with dyslipidemia, endothelial dysfunction, oxidative stress, and chronic inflammation, factors that are themselves associated with vascular and myocardial alterations. Similarly, lower intake of cardioprotective nutrients has been associated with less favorable vascular homeostasis. Therefore, evaluating the history of dietary patterns alongside documented clinical outcomes may help identify diet-related factors statistically associated with disease severity and adverse cardiovascular events. Collectively, these findings support the investigation of potential associations between dietary patterns and angiocardiopathy, providing a rationale for considering dietary strategies within comprehensive disease management and secondary prevention frameworks.

## 2 Methods

### 2.1 Study design and setting protocol

A retrospective cross-sectional study was conducted to assess the association between dietary risk factors and cardiovascular outcomes in patients with angiocardiopathy. Data were retrieved from electronic medical records, clinical databases, dietary intake assessments, and cardiovascular registries of several tertiary care hospitals between 1st January and 30th June 2025, thus providing a varied and representative patient population. This study was conducted in adherence to the STROBE (Strengthening the Reporting of Observational Studies in Epidemiology) guidelines for observational studies, and a completed checklist is provided as supplementary material.

### 2.2 Sample

The study included 525 adults diagnosed with angiocardiopathy, defined as clinically documented structural or functional cardiac and/or vascular disorders, including ischemic heart disease, heart failure, arrhythmias, or a history of revascularization procedures (PCI/CABG), confirmed from hospital electronic records. Patients were identified through convenience sampling. Inclusion criteria: (1) complete dietary intake records detailing macronutrients, micronutrients, sodium, and added sugars; (2) availability of calculated Dietary Inflammatory Index (China-DII) scores; and (3) documented cardiovascular disease, operationalized using ICD-10 codes I00–I99, with objective evidence of structural or functional cardiac abnormalities, such as echocardiographic findings (e.g., LVEF <50%, moderate-to-severe valvular lesions) or angiographic confirmation of coronary or peripheral vascular pathology or clinical documentation of cardiovascular outcomes, including acute coronary syndrome, stroke, major adverse cardiovascular events (MACE), cardiovascular hospitalization, or cardiovascular mortality. Exclusion criteria included congenital heart disease unrelated to angiocardiopathy, chronic systemic illnesses that significantly affect cardiovascular risk (e.g., end-stage renal or hepatic disease), and incomplete clinical or dietary records. As the study was based on pre-existing medical record data, dietary exposure and cardiovascular outcomes were assessed from available documentation to examine associations.

### 2.3 Data collection tool and procedure details

Data were extracted using a structured abstraction form developed based on relevant literature on cardiovascular nutrition and risk assessment. The form was reviewed by clinical nutrition and cardiology experts to ensure content validity, and a subset of records (10–15%) was independently extracted by two researchers to assess inter-rater reliability, with Cohen’s kappa used for categorical variables and intraclass correlation for continuous variables. The following variables were collected: Demographic and anthropometric characteristics: age, sex, height, weight, BMI, socioeconomic status, and residence. Clinical factors: hypertension, diabetes, dyslipidemia, smoking status, NYHA class, and left ventricular ejection fraction (LVEF). Dietary factors: Dietary intake data were obtained from patient records, which included diet diaries, 24-h recalls, food frequency questionnaires (FFQs), and dietitian notes in files. To enhance reliability, the FFQs and 24-h recalls used were previously validated in Chinese populations, and dietitians cross-checked entries to improve accuracy. Nutrient intake, including macronutrients, micronutrients, sodium, and added sugars, was calculated using the latest version of the Chinese Food Composition Tables (Standard Edition, 6th Edition, 2018), published by the National Institute for Nutrition and Health, Chinese Center for Disease Control and Prevention, ensuring inclusion of commonly consumed Chinese foods. Each food item was systematically mapped to the standard Dietary Inflammatory Index (China-DII) components following established procedures (17). Cardiovascular outcomes: any cardiovascular event, MACE, heart failure, acute coronary syndrome, arrhythmias, stroke, revascularization (PCI/CABG), cardiovascular hospitalization, and cardiovascular mortality.

### 2.4 Classification of dietary risk based on dietary inflammatory index (DII) score

Low Risk: Participants whose dietary intake was mostly aligned with recommended guidelines, adequate fruits, vegetables, whole grains, dietary fiber, omega-3 fatty acids, low added sugar, low saturated/trans fats, and lower sodium intake. Typically associated with a lower Dietary Inflammatory Index (DII) score (≤1.0). Moderate Risk: Participants with partially unhealthy dietary patterns, e.g., moderate intake of fruits/vegetables, higher refined carbohydrates, moderate added sugar, and slightly elevated saturated fat or sodium intake. Corresponds to a moderate DII score (1.1–2.9). High Risk: Participants with predominantly unhealthy dietary intake, low fruits/vegetables, high added sugar, high saturated/trans fats, excessive sodium, low fiber, and poor nutrient balance. Typically associated with a high DII score, indicating a pro-inflammatory diet (≥3.0).

Note: Complete dietary records were available in the patient files, allowing for accurate calculation of DII scores and assessment of dietary inflammatory potential.

### 2.5 Ethical considerations

The study protocol was approved by the Ethics Committee of the First Hospital of Qinhuangdao City (Approval No.2021C017), and was conducted according to the principles of the Declaration of Helsinki between January 2025 till June 2025. As the study involved a retrospective review of medical records, informed consent was waived. All patient identifiers were removed before analysis to maintain confidentiality.

### 2.6 Statistical analyses

Data were analyzed using SPSS 26. Continuous variables were summarized as mean ± SD or median (IQR), and categorical variables as counts and percentages. Group differences across dietary risk categories were assessed using ANOVA and *t*-tests for continuous variables and chi-square tests for categorical variables. Multivariable logistic regression was used to evaluate associations between dietary risk groups and cardiovascular outcomes, adjusting for age, sex, BMI, hypertension, diabetes, dyslipidemia, smoking, physical activity, and socioeconomic status were selected based on prior evidence identifying them as key confounders of diet–cardiovascular relationships, ensuring that associations were independent of these established risk factors. Multicollinearity among independent variables was assessed using the variance inflation factor (VIF), with VIF > 5 considered indicative of significant multicollinearity. Missing data were handled via multiple imputation under the assumption of missing at random, with five imputed datasets generated and pooled estimates reported. Adjusted odds ratios (ORs) with 95% CIs were reported, and significance was set at *p* < 0.05.

## 3 Results

### 3.1 Sociodemographic characteristics according to dietary risk groups

Table 1 presents notable differences in the socio-demographic characteristics of individuals across dietary risk groups, with pronounced quantitative trends. The progression of age followed dietary risk, the respective mean ages being 56.2 ± 11.8 years in the low-risk group, 57.9 ± 12.3 years in the moderate-risk group, and 60.1 ± 13.1 years in the high-risk group (*p* = 0.021). In line with this, the distribution of the age categories varied significantly (*p* = 0.032) as the participants aged 60 years made up 45.5% of the low-risk group, 49.0% of the moderate-risk group, and substantially rose to 69.9% in the high-risk group, while those <40 years dropped from 15.4% in the low-risk group to 7.2% in the high-risk group. Regarding gender, males constituted 58.0, 61.1, and 62.0% of the low, moderate, and high-risk groups, respectively, whereas females made up 42.0, 38.9, and 38.0%. Although males were dominant in all groups, the difference was not statistically significant (*p* = 0.078), thus, no significant sex, based disparity in dietary risk classification can be inferred. Educational status was the only socio-demographic factor showing a significant association with dietary risk (*p* = 0.015). Notably, the proportion of those with no formal education increased from 15.4% in the low-risk group to 30.6% in the moderate-risk group and 33.1% of the high-risk group. On the other hand, the proportions of persons with primary and secondary education were 52.4% in the low-risk group and 34.9% in the high-risk group, whereas higher education was the most prevalent in the low-risk group (32.2%) compared with the moderate and high-risk groups (25.0 and 31.9%, respectively). A strong inverse gradient could be discerned for income also (*p* = 0.009). People of low income accounted for 33.6% of the low-risk group, 47.2% of the moderate-risk group, and 54.8% of the high-risk group. Meanwhile, the participants with high income accounted for 23.8% of the low-risk group, 18.5% of the moderate risk group, and only 12.7% of the high-risk group, indicating a significant association between income level and dietary risk category. Furthermore, the residential status also showed a significant association with dietary risk group (*p* = 0.041), which varied across dietary risk groups. The proportion of urban residents was 71.3% in the low-risk group as compared to 61.1 and 62.0% in the moderate and high-risk groups, respectively, whereas those living in rural areas increased from 28.7% in the low-risk group to 38.9% in the moderate risk group and 38.0% in the high-risk group.

**Table 1:** Socio-demographic characteristics according to dietary risk groups.

Characteristic (*n* = 525)	Low risk (*n* = 143)	Moderate risk (*n* = 216)	High risk (*n* = 166)	*p*-value
Age (years), mean ± SD/median (IQR)	56.2 ± 11.8/55 (48–63)	57.9 ± 12.3/57 (50–65)	60.1 ± 13.1/60 (52–68)	0.021
Age category, *n* (%)				0.032
<40 years	22 (15.4)	28 (13.0)	12 (7.2)	
40–59 years	56 (39.2)	82 (38.0)	38 (22.9)	
≥60 years	65 (45.5)	106 (49.0)	116 (69.9)	
Sex, *n* (%)				0.078
Male	83 (58.0)	132 (61.1)	103 (62.0)	
Female	60 (42.0)	84 (38.9)	63 (38.0)	
Education, *n* (%)				0.015
No formal education	22 (15.4)	66 (30.6)	55 (33.1)	
Primary–secondary	75 (52.4)	96 (44.4)	58 (34.9)	
Higher education	46 (32.2)	54 (25.0)	53 (31.9)	
Income, *n* (%)				0.009
Low	48 (33.6)	102 (47.2)	91 (54.8)	
Middle	61 (42.7)	74 (34.3)	54 (32.5)	
High	34 (23.8)	40 (18.5)	21 (12.7)	
Residence, *n* (%)				0.041
Urban	102 (71.3)	132 (61.1)	103 (62.0)	
Rural	41 (28.7)	84 (38.9)	63 (38.0)	

Continuous variables are presented as mean ± SD/median (IQR). Normality was tested using the Shapiro–Wilk test. Categorical variables are presented as *n* (%). *p*-values were calculated using the chi-square test. A *p*-value < 0.05 was considered statistically significant.

### 3.2 Anthropometric characteristics according to dietary risk groups

As detailed in Table 2, anthropometric characteristics demonstrated significant differences across dietary risk groups, with adiposity indicators deteriorating alongside dietary risk escalation. Average height remained statistically similar across low, moderate, and high-risk groups (167.1 ± 8.3 cm, 166.5 ± 8.7 cm, and 165.6 ± 9.3 cm, respectively; *p* = 0.210). Meanwhile, body weight was raised steadily from 74.3 ± 12.5 kg in the low-risk group to 76.9 ± 13.0 kg in the moderate-risk group and finally to 79.5 ± 14.2 kg in the high-risk group (*p* = 0.008). The mean BMI illustrated a significant gradual increase across dietary risk groups, with values changing from 26.6 ± 4.4 kg/m in the low-risk group to 27.6 ± 4.7 kg/m in the moderate-risk group and 28.7 ± 5.2 kg/m in the high-risk group (*p* = 0.002). The BMI categories were additionally scrutinized to highlight this trend. The proportion of individuals with normal BMI dramatically decreased across dietary risk groups (41.3% in low risk, 36.1% in moderate risk, and 19.3% in high risk), while the percentages of overweight increased from 37.8 to 44.9 and 48.2%, and obesity increased from 21.0% in the low, risk group to 32.5% in the high, risk group (overall *p* = 0.011). Along with this, the measures of central obesity also changed significantly, as average waist circumference increased from 91.2 ± 10.5 cm in the low-risk group to 93.8 ± 11.2 cm in the moderate-risk group and 97.6 ± 12.1 cm in the high-risk group (*p* < 0.001). As a result, the prevalence of central obesity rose from 49.7% in the low-risk group to 58.8% in the moderate-risk group and 68.7% in the high-risk group (*p* = 0.003).

**Table 2:** Anthropometric characteristics according to dietary risk groups.

Parameter	Low risk	Moderate risk	High risk	*p*-value
Height (cm), mean ± SD/median (IQR)	167.1 ± 8.3/167 (160–174)	166.5 ± 8.7/166 (159–173)	165.6 ± 9.3165 (158–172)	0.210
Weight (kg), mean ± SD/median (IQR)	74.3 ± 12.5/74 (65–82)	76.9 ± 13.0/76 (68–85)	79.5 ± 14.2/78 (70–88)	0.008
BMI (kg/m^2^), mean ± SD/median (IQR)	26.6 ± 4.4/26 (23–30)	27.6 ± 4.7/27 (24–31)	28.7 ± 5.2/28 (25–33)	0.002
BMI category, *n* (%)				0.011
Normal	59 (41.3)	78 (36.1)	32 (19.3)	
Overweight	54 (37.8)	97 (44.9)	80 (48.2)	
Obese	30 (21.0)	41 (19.0)	54 (32.5)	
Waist circumference (cm), mean ± SD/median (IQR)	91.2 ± 10.5/91 (84–98)	93.8 ± 11.2/94 (86–101)	97.6 ± 12.1/97 (89–105)	<0.001
Central obesity, *n* (%)	71 (49.7)	127 (58.8)	114 (68.7)	0.003

Continuous variables are presented as mean ± SD/median (IQR). Normality was tested using the Shapiro–Wilk test. Categorical variables are presented as *n* (%). *p*-values were calculated using the chi-square test. A *p*-value < 0.05 was considered statistically significant.

### 3.3 Clinical characteristics according to dietary risk groups

As presented in Table 3, several adverse clinical characteristics were more prevalent among participants with higher dietary risk. The prevalence of hypertension increased significantly across dietary risk groups, affecting 54.5% of individuals in the low-risk group, 62.0% in the moderate-risk group, and 71.7% in the high-risk group (*p* = 0.005). Similarly, diabetes mellitus was more common with increasing dietary risk, rising from 39.9% in the low-risk group to 49.1% in the moderate-risk group and 50.0% in the high-risk group (*p* = 0.047). Smoking prevalence also differed significantly across groups, with 26.6% of low-risk participants reporting smoking compared with 38.0 and 37.3% in the moderate- and high-risk groups, respectively (*p* = 0.028). Markers of disease severity showed a clear gradient with dietary risk. A higher proportion of participants in the moderate- and high-risk groups were classified as NYHA functional class III–IV, increasing from 27.3% in the low-risk group to 36.1% in the moderate-risk group and 47.6% in the high-risk group (*p* = 0.001). Left ventricular ejection fraction (LVEF) progressively decreased with increasing dietary risk in a very consistent manner. Mean values of LVEF were 48.1 ± 11.2% in the low risk group, 45.3 ± 11.5% in the moderate risk group, and 43.0 ± 12.2% in the high risk group (*p* < 0.001), thus, poorer cardiac function was found in those with worse dietary patterns. Dyslipidemia, on the other hand, showed a non-significant trend towards higher prevalence in the moderate and high-risk groups (49.0, 8.8, and 58.4%, respectively; *p* = 0.082).

**Table 3:** Clinical characteristics according to dietary risk groups.

Characteristic	Low risk	Moderate risk	High risk	*p*-value
Hypertension, *n* (%)	78 (54.5)	134 (62.0)	119 (71.7)	0.005
Diabetes mellitus, *n* (%)	57 (39.9)	106 (49.1)	83 (50.0)	0.047
Dyslipidemia, *n* (%)	70 (49.0)	127 (58.8)	97 (58.4)	0.082
Smoking, *n* (%)	38 (26.6)	82 (38.0)	62 (37.3)	0.028
NYHA class III–IV, *n* (%)	39 (27.3)	78 (36.1)	79 (47.6)	<0.001
LVEF (%), mean ± SD/median (IQR)	48.1 ± 11.2/48 (40–55)	45.3 ± 11.5/45 (37–53)	43.0 ± 12.2/42 (35–50)	<0.001

Continuous variables are presented as mean ± SD/median (IQR). Normality was tested using the Shapiro–Wilk test. Categorical variables are presented as *n* (%). *p*-values were calculated using the chi-square test. A *p*-value < 0.05 was considered statistically significant.

### 3.4 Dietary intake characteristics according to dietary risk groups

The characteristics of dietary intake differed significantly between the dietary risk groups, as progressive worsening of diet quality with increasing risk was clearly visible (Table 4). Total energy intake was increasing in a stepwise manner from 2,058 ± 318 kcal/day in the low, risk group to 2,237 ± 392 kcal/day in the moderate, risk group and 2,471 ± 425 kcal/day in the high, risk group (*p* < 0.001). The percentage of energy derived from carbohydrates increased slightly in the groups (51.2 to 53.7%; *p* = 0.017), while the protein contribution decreased significantly from 18.5% in the low-risk group to 16.4% in the high-risk group (*p* < 0.001). Total fat intake increased from 29.3 to 31.5% of total energy (*p* = 0.008), where the increase in saturated fat was particularly prominent (8.6% vs. 14.8% of energy), as well as the increase in trans-fat intake (1.2% vs. 2.6% of energy) from low, to high, risk groups (both *p* < 0.001). Dietary cholesterol intake also increased significantly, from 245 ± 72 mg/day in the low-risk group to 401 ± 102 mg/day in the high-risk group (*p* < 0.001). On the other hand, protective dietary elements demonstrated a steady decrease as dietary risk increased. The intake of dietary fiber was reduced from 28.4 ± 5.6 g/day in the low-risk group to 21.2 ± 4.8 g/day in the moderate-risk group and 14.6 ± 3.9 g/day in the high-risk group (*p* < 0.001). The consumption of fruits and vegetables also mirrored this trend, with fruit intake decreasing from 2.8 ± 1.1 to 1.2 ± 0.8 servings/day and vegetable intake from 2.1 ± 0.9 to 0.9 ± 0.5 servings/day, respectively, across different risk categories (both *p* < 0.001). The intakes of omega-3 fatty acids (1.2 ± 0.5 vs. 0.6 ± 0.3 g/day), calcium (756 ± 134 vs. 612 ± 115 mg/day), and vitamin E (10.4 ± 2.8 vs. 6.9 ± 2.1 mg/day) also decreased significantly from the low- to high-risk groups (all *p* < 0.001). On the other hand, higher-risk diets were mainly associated with an excessive consumption of various harmful components. Sodium intake went up significantly from 2,110 ± 420 mg/day in the low-risk group to 3,560 ± 640 mg/day in the high-risk group (*p* < 0.001), while added sugar intake increased from 28.3 ± 12.5 to 58.4 ± 17.2 g/day (*p* < 0.001). The consumption of sugar, sweetened beverages was more than tripled across dietary risk categories (0.5 ± 0.3 to 1.8 ± 0.7 servings/day; *p* < 0.001). The Dietary Inflammatory Index (DII) score also followed these trends and increased significantly from 0.8 ± 0.6 in the low-risk group to 3.4 ± 1.1 in the high-risk group (*p* < 0.001), thereby pointing to a gradually more pro-inflammatory dietary profile.

**Table 4:** Dietary intake characteristics according to dietary risk groups.

Dietary variable (*n* = 525)	Low risk (*n* = 143)	Moderate risk (*n* = 216)	High risk (*n* = 166)	*p*-value
Total energy intake (kcal/day)/median (IQR)	2,058 ± 318/2,050 (1,850–2,240)	2,237 ± 392/2,240 (2,000–2,450)	2,471 ± 425/2,480 (2,200–2,700)	<0.001
Carbohydrate (% energy)/median (IQR)	51.2 ± 6.9/51 (46–57)	52.6 ± 7.3/53 (48–58)	53.7 ± 8.2/54 (48–60)	0.017
Protein (% energy)/median (IQR)	18.5 ± 3.4/18 (16–21)	17.7 ± 3.8/18 (15–21)	16.4 ± 4.1/16 (13–19)	<0.001
Total fat (% energy)/median (IQR)	29.3 ± 5.2/29 (25–34)	30.2 ± 6.0/30 (25–35)	31.5 ± 6.7/32 (26–37)	0.008
Saturated fat (% energy)/median (IQR)	8.6 ± 1.9/9 (7–10)	11.4 ± 2.1/11 (10–13)	14.8 ± 2.6/15 (12–17)	<0.001
Trans-fat (% energy)/median (IQR)	1.2 ± 0.4/1.2 (1.0–1.5)	1.9 ± 0.6/1.8 (1.5–2.2)	2.6 ± 0.7/2.5 (2.0–3.0)	<0.001
Dietary cholesterol (mg/day)/median (IQR)	245 ± 72/240 (190–300)	312 ± 91/310 (250–370)	401 ± 102/400 (330–470)	<0.001
Dietary fiber (g/day)/median (IQR)	28.4 ± 5.6/28 (24–32)	21.2 ± 4.8/21 (17–25)	14.6 ± 3.9/15 (12–18)	<0.001
Fruit intake (serv/day)/median (IQR)	2.8 ± 1.1/3 (2–4)	2.1 ± 1.0/2 (1–3)	1.2 ± 0.8/1 (0.5–2)	<0.001
Vegetable intake (serv/day)/median (IQR)	2.1 ± 0.9/2 (1–3)	1.6 ± 0.8/1.5 (1–2)	0.9 ± 0.5/1 (0.5–1)	<0.001
Sodium intake (mg/day)/median (IQR)	2,110 ± 420/2,100 (1,800–2,400)	2,840 ± 510/2,850 (2,400–3,200)	3,560 ± 640/3,550 (3,100–4,000)	<0.001
Added sugar intake (g/day)/median (IQR)	28.3 ± 12.5/27 (18–38)	41.7 ± 14.6/42 (30–55)	58.4 ± 17.2/58 (45–70)	<0.001
Sugar-sweetened beverages (serv/day)/median (IQR)	0.5 ± 0.3/0.5 (0.3–0.7)	1.1 ± 0.5/1.0 (0.7–1.5)	1.8 ± 0.7/1.8 (1.3–2.3)	<0.001
Omega-3 fatty acids (g/day)/median (IQR)	1.2 ± 0.5/1.2 (0.8–1.5)	0.9 ± 0.4/0.9 (0.6–1.2)	0.6 ± 0.3/0.6 (0.4–0.8)	<0.001
Calcium (mg/day)/median (IQR)	756 ± 134/750 (650–840)	693 ± 128/690 (600–780)	612 ± 115/610 (520–700)	<0.001
Vitamin E (mg/day)/median (IQR)	10.4 ± 2.8/10 (8–13)	8.7 ± 2.4/9 (7–11)	6.9 ± 2.1/7 (5–9)	<0.001
Dietary Inflammatory Index (DII) score/median (IQR)	0.8 ± 0.6/0.8 (0.3–1.3)	2.0 ± 0.9/2.0 (1.3–2.7)	3.4 ± 1.1/3.5 (2.5–4.2)	<0.001

Continuous variables are presented as mean ± SD/median (IQR). Normality was tested using the Shapiro–Wilk test. Categorical variables are presented as *n* (%). *p*-values were calculated using the chi-square test. A *p*-value < 0.05 was considered statistically significant.

### 3.5 Cardiovascular outcomes according to dietary risk groups

Data presented in Table 5 illustrate stepwise changes in the prevalence of cardiovascular outcomes among dietary risk groups, thereby emphasizing the relationship between worsening dietary patterns and adverse cardiovascular health. Any cardiovascular event was documented in 37.8% of subjects from the low-risk group, 44.4% in the moderate-risk group, and 73.5% in the high-risk group, with the trend being statistically highly significant (*p* < 0.001). Likewise, major adverse cardiovascular events (MACE) accounted for 27.3% of the low-risk individuals, 38.0% of the moderate-risk group, and 54.2% of the high-risk dietary risk group, respectively (*p* < 0.001). The prevalence of heart failure also manifested a notable stepwise change, with its rate rising from 28.7% in the low-risk group, 39.4% in the moderate-risk group, and 53.0% in the high-risk group (*p* < 0.001). On the other hand, ischemic heart disease was prevalent in all groups (50.3, 54.6, and 59.6% in the low, moderate, and high-risk groups, respectively); however, the difference was not statistically significant (*p* = 0.193). Acute coronary syndrome showed a significant increase, going from 17.5% in the low-risk group of patients, 27.8% in the moderate-risk group, and 34.9% in the high-risk group (*p* = 0.002). Arrhythmias were found in 26.6% of participants with low dietary risk, increasing slightly to 29.6% in the moderate-risk group and considerably to 44.6% in the high-risk group (*p* = 0.003). Stroke prevalence was one of the strongest gradients, increasing from 7.7% in the low-risk group to 14.8% in the moderate-risk group and 32.5% in the high-risk group (*p* < 0.001). Similarly, the requirement for revascularization procedures (PCI/CABG) was augmented significantly across dietary risk categories, from 22.4% in the low-risk group to 30.6% in the moderate-risk group and 39.8% in the high-risk group (*p* = 0.006). Hospitalizations due to cardiovascular disease also showed a similar trend, representing 36.4% of the low-risk participants, 46.8% of the moderate-risk participants, and 65.1% of the high-risk participants (*p* < 0.001). Most notably, death from cardiovascular causes went up by more than three times as the dietary risk categories worsened, going from 6.3% in the low-risk group to 10.2% in the moderate-risk group and 22.3% in the high-risk group (*p* < 0.001).

**Table 5:** Cardiovascular outcomes according to dietary risk groups.

Cardiovascular outcome (*n* = 525)	Low risk (*n* = 143)	Moderate risk (*n* = 216)	High risk (*n* = 166)	*p*-value
Any cardiovascular event	54 (37.8%)	96 (44.4%)	122 (73.5%)	<0.001
Major adverse cardiovascular events (MACE)	39 (27.3%)	82 (38.0%)	90 (54.2%)	<0.001
Heart failure	41 (28.7%)	85 (39.4%)	88 (53.0%)	<0.001
Ischemic heart disease	72 (50.3%)	118 (54.6%)	99 (59.6%)	0.193
Acute coronary syndrome	25 (17.5%)	60 (27.8%)	58 (34.9%)	0.002
Arrhythmias	38 (26.6%)	64 (29.6%)	74 (44.6%)	0.003
Stroke	11 (7.7%)	32 (14.8%)	54 (32.5%)	<0.001
Revascularization (PCI/CABG)	32 (22.4%)	66 (30.6%)	66 (39.8%)	0.006
Cardiovascular hospitalization	52 (36.4%)	101 (46.8%)	108 (65.1%)	<0.001
Cardiovascular mortality	9 (6.3%)	22 (10.2%)	37 (22.3%)	<0.001

Values are presented as *n* (%). *p*-values were derived using the chi-square test for categorical variables to compare cardiovascular outcomes across dietary risk groups. A *p*-value < 0.05 was considered statistically significant.

### 3.6 Multivariable logistic regression analysis of dietary risk groups and cardiovascular outcomes (*n* = 525)

Multivariable logistic regression analysis, shown in Table 6, revealed a clear gradient between dietary risk and cardiovascular outcomes along the entire spectrum from the low-risk dietary group that was used as the reference. In comparison to this group, those categorized as moderate risk had increased odds for most outcomes, yet, for several associations, the differences did not attain statistical significance. Moderate risk diet was linked to a 28% increase in the odds of any cardiovascular event (AOR = 1.28, 95% CI: 0.871.88; *p* = 0.21), a 41% increase in the odds of major adverse cardiovascular events (MACE) (AOR = 1.41, 95% CI: 1.021.96; *p* = 0.038), a 49% increase in the odds of heart failure (AOR = 1.49, 95% CI: 0.982.27; *p* = 0.061), a 75% increase in the odds of acute coronary syndrome (AOR = 1.75, 95% CI: 1.013.02; *p* = 0.045), a twofold increase in the odds of stroke (AOR = 2.01, 95% CI: 0.954.25; *p* = 0.066), and a 62% increase in the odds of cardiovascular mortality (AOR = 1.62, 95% CI: 0.713.69; *p* = 0.244). Participants in the high-risk dietary group, on the other hand, showed consistently strong and statistically significant associations across all outcomes. They had three times higher odds of any cardiovascular event (AOR = 3.01, 95% CI: 1.924.72; *p* < 0.001), 2.63 times higher odds of MACE (AOR = 2.63, 95% CI: 1.813.83; *p* < 0.001), more than two times higher odds of heart failure (AOR = 2.23, 95% CI: 1.403.55; *p* < 0.001), 2.54 times higher odds of acute coronary syndrome (AOR = 2.54, 95% CI: 1.444.47; *p* = 0.002), nearly five times higher odds of stroke (AOR = 4.82, 95% CI: 2.419.64; *p* < 0.001), and 4.13 times higher odds of cardiovascular mortality (AOR = 4.13, 95% CI: 1.789.57; *p* < 0.001).

**Table 6:** Multivariable logistic regression analysis of dietary risk groups and cardiovascular outcomes.

Outcome (*n* = 525)	Dietary risk group	*β* (SE)	Adjusted OR (95% CI)	*p*-value
Any cardiovascular event	Low risk	Reference	Reference	–
	Moderate risk	0.25 (0.15)	1.28 (0.87–1.88)	0.21
	High risk	1.10 (0.23)	3.01 (1.92–4.72)	<0.001
MACE	Low risk	Reference	Reference	–
	Moderate risk	0.34 (0.16)	1.41 (1.02–1.96)	0.038
	High risk	0.97 (0.21)	2.63 (1.81–3.83)	<0.001
Heart failure	Low risk	Reference	Reference	–
	Moderate risk	0.40 (0.21)	1.49 (0.98–2.27)	0.061
	High risk	0.80 (0.25)	2.23 (1.40–3.55)	<0.001
Acute coronary syndrome	Low risk	Reference	Reference	–
	Moderate risk	0.56 (0.31)	1.75 (1.01–3.02)	0.045
	High risk	0.93 (0.32)	2.54 (1.44–4.47)	0.002
Stroke	Low risk	Reference	Reference	–
	Moderate risk	0.70 (0.37)	2.01 (0.95–4.25)	0.066
	High risk	1.57 (0.36)	4.82 (2.41–9.64)	<0.001
Cardiovascular mortality	Low risk	Reference	Reference	–
	Moderate risk	0.48 (0.42)	1.62 (0.71–3.69)	0.244
	High risk	1.42 (0.47)	4.13 (1.78–9.57)	<0.001

Multivariable logistic regression models were used to calculate adjusted odds ratios (ORs) and 95% confidence intervals (CIs). Models were adjusted for age, sex, BMI, hypertension, diabetes, dyslipidemia, smoking, physical activity, and socioeconomic status, consistent with the covariates specified in the Methods section. Statistical significance was set at *p* < 0.05.

## 4 Discussion

This study evaluates the association between dietary risk profiles and adverse cardiovascular outcomes in patients with angiocardiopathy using a retrospective observational design. Table 1 shows that participants with high dietary risk were older (mean age 60.1 ± 13.1 vs. 56.2 ± 11.8 years; *p* = 0.021), had lower educational attainment (33.1% vs. 15.4% with no formal education; *p* = 0.015), lower income (54.8% vs. 33.6% low income; *p* = 0.009), and were more often rural residents (38.0% vs. 28.7%; *p* = 0.041). These socio-demographic disparities were associated with differences in dietary patterns and cardiovascular risk. Lower levels of education and health literacy may be linked to reduced adherence to cardioprotective dietary patterns (18, 19). Individuals with lower income living in a rural area may have limited access to fresh fruits, vegetables, whole grains, and lean proteins, which results in the consumption of diets high in saturated fats, added sugars, and sodium, such dietary profiles have been associated with endothelial dysfunction, dyslipidemia, hypertension, and chronic low-grade inflammation, which are recognized contributors to atherosclerotic progression (20–22). Moreover, sociodemographic disadvantages often correlated with clustering of behavioral and psychosocial risk factors such as smoking prevalence, reduced healthcare access, and stress, related metabolic effects (23). Importantly, these findings have implications for the feasibility of implementing dietary interventions in older patients with multiple comorbidities. Advanced age and multimorbidity may be associated with reduced functional capacity, mobility limitations, and decreased independence in food selection and preparation, which in turn may be linked to lower adherence to prescribed dietary modifications. Financial constraints and rural residence may be associated with limited availability and affordability of healthy food options, potentially influencing sustained dietary change. Similarly, low educational attainment and limited health literacy may be associated with reduced understanding of nutritional recommendations and long-term compliance.

Table 2 presents anthropometric profiles that are associated with increasingly worse outcomes as the dietary risk increases. It also reveals a very strong correlation between pro-inflammatory dietary patterns and adiposity markers. Mean height was not significantly different (167.1 ± 8.3 cm in low risk vs. 165.6 ± 9.3 cm in high risk, *p* = 0.210); however, weight increased significantly from 74.3 ± 12.5 kg in the low-risk group to 79.5 ± 14.2 kg in the high-risk group (*p* = 0.008). This resulted in higher mean BMI values (28.7 ± 5.2 kg/m in high risk vs. 26.6 ± 4.4 kg/m in low risk, *p* = 0.002), and the prevalence of overweight and obesity increased. Only 19.3% of high-risk participants had a normal BMI compared to 41.3% of low-risk participants (*p* = 0.011), while obesity was the most frequent in the high-risk group (32.5% vs. 21.0%). Waist circumference, a well-known measure of visceral adiposity, also showed a stepwise increase (97.6 ± 12.1 cm in high risk vs. 91.2 ± 10.5 cm in low risk, *p* < 0.001), and central obesity was more prevalent in the high-risk group (68.7% vs. 49.7%, *p* = 0.003). These findings are consistent with previous studies that have identified a significant association between pro-inflammatory diets and increased adiposity. Ozen et al. similarly reported that diets high in saturated fats, processed carbohydrates, and added sugars were associated with an increase in visceral fat, which in turn was associated with metabolic syndrome components (waist circumference ~98 ± 11 cm vs. 88 ± 9 cm for healthier diets; *p* < 0.01) (24, 25). Central obesity is strongly associated with cardiovascular risk by creating a pro-atherogenic and pro-thrombotic milieu characterized by dyslipidemia, endothelial dysfunction, and increased oxidative stress (26, 27). Hence, the anthropometric changes recorded reveal the interaction between dietary quality, adiposity, and the burden of cardiovascular risk in angiocardiopathy.

Table 3 reveals a staggered deterioration of clinical risk profiles across dietary risk groups, indicating that a higher dietary risk is associated with a greater number of established cardiovascular risk factors and increased disease severity. The prevalence of hypertension significantly increased from 54.5% in the low-risk group to 71.7% in the high-risk group (*p* = 0.005), while diabetes mellitus was also more frequent in moderate and high-risk groups (49.1 and 50.0%, respectively, vs. 39.9%; *p* = 0.047). The prevalence of dyslipidemia tended to increase, but the differences between groups were not statistically significant (49.0% in low risk vs. 58.8% in moderate and 58.4% in high risk; *p* = 0.082). Smoking prevalence was higher in the moderate and high-risk groups (38.0 and 37.3% vs. 26.6%; *p* = 0.028). More importantly, the severity of functional limitation as measured by NYHA class IIIIV was progressively more common from low (27.3%) to high dietary risk (47.6%; *p* = 0.001). Left ventricular ejection fraction (LVEF) also decreased stepwise (48.1 ± 11.2% in low risk vs. 43.0 ± 12.2% in high risk; *p* < 0.001), indicating worse cardiac function associated with higher dietary risk. These findings are in line with previous studies that have linked pro-inflammatory diets to clinical cardiovascular risk. An example of this is a large prospective cohort which showed that individuals in the highest third of the Dietary Inflammatory Index (DII) had significantly higher odds of hypertension and type 2 diabetes compared to those in the lowest third (ORs 1.30 1.45; *p* < 0.01) even after adjusting for confounders, indicating that diet-induced inflammation is associated with metabolic dysregulation and elevated blood pressure (28). Likewise, a cross-sectional analysis of cardiac patients found that higher intake of saturated fats and refined carbohydrates was associated with a 1.5-fold higher prevalence of smoking and unhealthy lifestyle clustering, thereby increasing cardiovascular risk and decreasing functional status (29–31). Hence, it strongly supports the need for dietary assessment to be considered not only as a tool for cardiovascular risk stratification but also as a key element in its management.

Table 4 exhibits profound and stepwise differences in food consumption that vary distinctly among dietary risk groups and align with the known cardio-metabolic risk pathways. Total energy intake escalated dramatically and significantly from 2,058 ± 318 kcal/day in the low-risk group to 2,471 ± 425 kcal/day in the high-risk group (*p* < 0.001), which is consistent with the description of energy-dense dietary patterns in the higher-risk strata. Carbohydrate intake as a percentage of energy also changed slightly (51.2 ± 6.9% to 53.7 ± 8.2%, *p* = 0.017), with protein (% energy) reducing (18.5 ± 3.4% to 16.4 ± 4.1%, *p* < 0.001), indicating a shift toward lower protein quality. Of significance, total fat (% energy) went up (29.3 ± 5.2% vs. 31.5 ± 6.7%, *p* = 0.008), while saturated fat increased highly from 8.6 1.9% in the low-risk group to 14.8 2.6% in the high-risk group (*p* < 0.001), and trans fat followed a similar pattern (1.2 ± 0.4% vs. 2.6 ± 0.7%, *p* < 0.001). Besides that, dietary cholesterol (245 ± 72 mg to 401 ± 102 mg, *p* < 0.001) and sodium intake (2,110 ± 420 mg to 3,560 ± 640 mg, *p* < 0.001) were also the highest among high-risk individuals. Protective nutrients and foods also followed the same patterns as the other variables and were significantly lower in high-risk groups: dietary fiber decreased from 28.4 ± 5.6 g/day to 14.6 ± 3.9 g/day (*p* < 0.001), fruit intake from 2.8 ± 1.1 to 1.2 ± 0.8 servings/day (*p* < 0.001), and vegetable intake from 2.1 ± 0.9 to 0.9 ± 0.5 servings/day (*p* < 0.001). Omega-3 fatty acids declined from 1.2 ± 0.5 g/day to 0.6 ± 0.3 g/day (*p* < 0.001), and micronutrients such as calcium and vitamin E were significantly lower in the high-risk group (all *p* < 0.001). These differences mirrored the changes in Dietary Inflammatory Index (DII) scores, which increased from 0.8 ± 0.6 in low risk to 3.4 ± 1.1 in high risk (*p* < 0.001), corresponding to more pro-inflammatory dietary exposures. These results are consistent with those from the Multi Ethnic Study of Atherosclerosis (MESA), where individuals in the highest quintile of saturated fat intake (1,516% of energy) had 1.6-fold higher risk of coronary events compared with those in the lowest quintile (89% of energy), independent of traditional risk factors (*p* < 0.01). Similarly, Brady et al. reaffirmed that diets with a sodium content of more than 2,500 mg per day were associated with 1.4 times the risk of hypertension and cardiovascular events compared to lower sodium intake (less than 2,000 mg per day) (*p* < 0.05), thus pointing out the influence of excess sodium on blood pressure and vascular function (32, 33). Table 5 displays a gradual association between dietary risk patterns and cardiovascular outcomes in patients with angiocardiopathy. The frequency of any cardiovascular event escalated significantly from 37.8% in the low-risk group to 73.5% in the high-risk group (*p* < 0.001). Likewise, major adverse cardiovascular events (MACE) were considerably higher in the high-risk group (54.2%) compared to the moderate (38.0%) and low-risk groups (27.3%, *p* < 0.001). The rate of heart failure increased from 28.7% in low-risk to 53.0% in high-risk (*p* < 0.001), and acute coronary syndrome was present in 34.9% of high-risk patients compared to 17.5% in low-risk (*p* = 0.002). Moreover, arrhythmias (44.6% vs. 26.6%, *p* = 0.003), stroke (32.5% vs. 7.7%, *p* < 0.001), revascularization procedures (39.8% vs. 22.4%, *p* = 0.006), cardiovascular hospitalization (65.1% vs. 36.4%, *p* < 0.001), and cardiovascular mortality (22.3% vs. 6.3%, *p* < 0.001) were all significantly elevated in the high dietary risk group. The present data are consistent with and extend previous findings linking pro-inflammatory and nutrient-poor diets to adverse cardiovascular outcomes. The non-significant association between dietary risk and ischemic heart disease (*p* = 0.193) may be influenced by stronger established risk factors, including hypertension, diabetes, and prior cardiac injury. Additionally, the presence of competing cardiovascular outcomes may attenuate the observed association between diet and IHD in this population. Kirkpatrick et al. (2018) provided an example that individuals consuming high saturated fat and low fiber diets had twice to thrice the risk of MACE and heart failure, even after accounting for typical risk factors (34). In a high-fat situation, the consumption of saturated fats, trans fats, and sodium causes endothelial dysfunction, raises LDL cholesterol, and initiates oxidative stress, while the overconsumption of added sugars worsens insulin resistance and chronic inflammation. Deficiencies in fiber, fruits, vegetables, and omega-3 fatty acids reduce the body’s anti-inflammatory defenses, lower nitric oxide availability, and speed up atherogenesis (35, 36). Table 6 displays the results of a multivariable logistic regression analysis that evaluated the independent association of dietary risk groups with cardiovascular outcomes. The analysis controlled for confounders such as age, sex, BMI, hypertension, diabetes, dyslipidemia, smoking, and socioeconomic status. A significantly high dietary risk was associated with increased odds of adverse cardiovascular outcomes for several endpoints. In particular, the subjects with the highest risk diet were associated with a threefold increase in odds of any cardiovascular event (OR 3.01, 95% CI 1.924.72, *p* < 0.001). Likewise, the risk of major adverse cardiovascular events (MACE) escalated by 2.63 times (OR 2.63, 95% CI 1.813.83, *p* < 0.001), heart failure by 2.23 times (OR 2.23, 95% CI 1.403.55, *p* < 0.001), and acute coronary syndrome by 2.54 times (OR 2.54, 95% CI 1.444.47, *p* = 0.002). The most substantial risk increases were observed in stroke and cardiovascular mortality, whose ORs were 4.82 (95% CI 2.419.64, *p* < 0.001) and 4.13 (95% CI 1.789.57, *p* < 0.001), respectively. Contrastingly, moderate dietary risk was linked to smaller and mostly non-significant odds of cardiovascular risk, except for MACE (OR 1.41, 95% CI 1.021.96, *p* = 0.038) and acute coronary syndrome (OR 1.75, 95% CI 1.013.02, *p* = 0.045), which implies a dose-dependent association between dietary quality and cardiovascular outcomes. In fact, high-risk dietary patterns over consumption of saturated and trans fats, added sugar, and sodium, with a low intake of fiber, fruits, vegetables, and omega-3 fatty acids, are associated with systemic inflammation, endothelial dysfunction, dyslipidemia, and oxidative stress, hence speeding the process of atherosclerosis and myocardial remodeling (37, 38). The high-risk group (3.4 ± 1.1) had elevated Dietary Inflammatory Index (DII) scores, which is indicative of a pro-inflammatory diet. This has been directly linked to increased incidents of stroke, heart failure, and cardiovascular mortality in the population, based on studies (39, 40). These results conform to the independent and stair-like effect of diet quality on cardiovascular prognosis in angiocardiopathy, emphasizing the necessity of dietary interventions for secondary prevention. Overall, our results support existing evidence that pro-inflammatory diets, indicated by higher DII scores, increase cardiovascular risk, consistent with findings from previous studies (11, 41, 42), while acknowledging that studies like PURE (43) highlight variability and complexity in nutritional epidemiology.

### 4.1 Strengths

This research evaluated how long-term diets, measured through the Dietary Inflammatory Index (DII), relate to several cardiovascular outcomes in patients with angiocardiopathy. It found, with a substantial multi-center cohort and comprehensive dietary, clinical, and socio-demographic data, an association of pro-inflammatory diets with elevated cardiovascular risk.

### 4.2 Limitations

Although conducted across multiple hospitals, the cross-sectional design and convenience sampling may introduce selection bias, potentially overrepresenting patients with more complete dietary records or frequent healthcare visits. Additionally, the study’s regional context may limit the generalizability of the findings to other populations. We also did not conduct subgroup analyses (e.g., by sex, age, or diabetes status) or sensitivity analyses (e.g., altering DII cutoffs) to test the robustness of the findings. Furthermore, as a retrospective observational study, causality cannot be inferred, and residual confounding from unmeasured variables may still exist.

### 4.3 Conclusion

This retrospective analysis illustrates in great detail how diet, related risk, is significantly associated with progressively worsening cardiovascular outcomes in patients with angio-cardiopathy. Those individuals following a high, risk, pro, inflammatory diet were notable for having a more negative socio, demographic profile, increased measures of adiposity, worsened metabolic and clinical status, and a markedly higher occurrence of cardiovascular events such as MACE (54.2%), stroke (32.5%), and cardiovascular mortality (22.3%) when compared to those at low risk. The current evidence very clearly underscores that diet, diet-related inflammation, and overconsumption of saturated fat and sodium, and insufficient intake of protective nutrients are associated with the progression of vascular and myocardial abnormalities. Prospective and interventional studies are required to establish causality and to assess whether anti-inflammatory, cardioprotective dietary interventions can lower the incidence of cardiovascular events and improve long-term outcomes in patients with angiocardiopathy.

## Data Availability

The original contributions presented in the study are included in the article/supplementary material, further inquiries can be directed to the corresponding author.
